# A Vanishing Clot: The Disappearance of a Free‐Floating Right Atrial Thrombus Just Before Urgent Surgical Thrombectomy

**DOI:** 10.1155/crcc/9997423

**Published:** 2025-12-29

**Authors:** Emanuel Heil, Jan Matthias Kruse, Daniel Zickler, Christoph Starck, Petar Petrov, Julius Valentin Kunz

**Affiliations:** ^1^ Department of Cardiology, Angiology and Intensive Care Medicine, Deutsches Herzzentrum der Charité (DHZC), Berlin, Germany; ^2^ Department of Nephrology and Medical Intensive Care, Charité-Universitätsmedizin Berlin, Freie Universität Berlin, and Humboldt-Universität zu Berlin, Berlin Institute of Health, Berlin, Germany, fu-berlin.de; ^3^ DZHK (German Centre for Cardiovascular Research) Partner Site Berlin, Berlin, Germany; ^4^ Department of Nephrology and Medical Intensive Care, St. Joseph Hospital, Berlin, Germany, stjosephhospital.com; ^5^ Department of Cardiothoracic and Vascular Surgery, Deutsches Herzzentrum der Charité (DHZC), Berlin, Germany

**Keywords:** point-of care-ultrasound, pulmonary embolism, right atrial thrombus, surgical thrombectomy

## Abstract

We present the case of a 68‐year‐old male who experienced presyncope and was found to have a large, free‐floating thrombus in the right atrium, accompanied by a concurrent pulmonary embolism. Given the high risk of a massive pulmonary embolism, a multidisciplinary team recommended surgical thrombectomy under cardiopulmonary bypass. However, shortly after the treatment decision, the thrombus spontaneously disappeared, most likely having embolized into the pulmonary arteries. As the patient exhibited minimal hemodynamic compromise, the team opted for thrombolytic therapy with rt‐PA instead of surgery. The patient was discharged 10 days later following an uneventful recovery from infarct pneumonia. This case highlights the unpredictable behavior of intracardiac thrombi and emphasizes the importance of ongoing reassessment and imaging in guiding clinical management.

## 1. Introduction

Large right atrial thrombi are rare but can present life‐threatening complications such as acute right heart failure, particularly when free‐floating. Since there is no established consensus on optimal treatment, management decisions are typically individualized and guided by multidisciplinary risk–benefit evaluation [1, 2]. Commonly employed methods are pharmacological thrombolysis [3, 4], interventional catheter‐based thrombus aspiration [5, 6], or surgical thrombectomy [7]. In the management of this patient, we encountered an unexpected resolution or mobilization of a large right atrial thrombus, which vanished spontaneously before surgery, forcing a rapid shift in the treatment strategy.

## 2. Case Presentation and Point‐of‐Care Investigations

A relatively lean 68‐year‐old male was presented to the emergency department with complaints of dizziness, lightheadedness, and orthostatic presyncope symptoms but remained asymptomatic while lying down or sitting. Upon arrival, he was hemodynamically stable with a heart rate of 90 bpm, blood pressure of 130/78 mmHg, a mildly elevated respiratory rate of 18 breaths per minute, and a peripheral oxygen saturation (SpO_2_) of 94% at rest. There were no clinical signs of deep vein thrombosis (DVT), fever, altered mental status, or hemoptysis. His medical history was notable for obstructive sleep apnea (OSA) with poor adherence to continuous positive airway pressure (CPAP) therapy, a left‐sided DVT three years prior, and a central pulmonary embolism complicated by infarct pneumonia during the same year. He had been on therapeutic anticoagulation, which was discontinued one year ago after a prolonged period without recurrent events.

**Figure 1:**
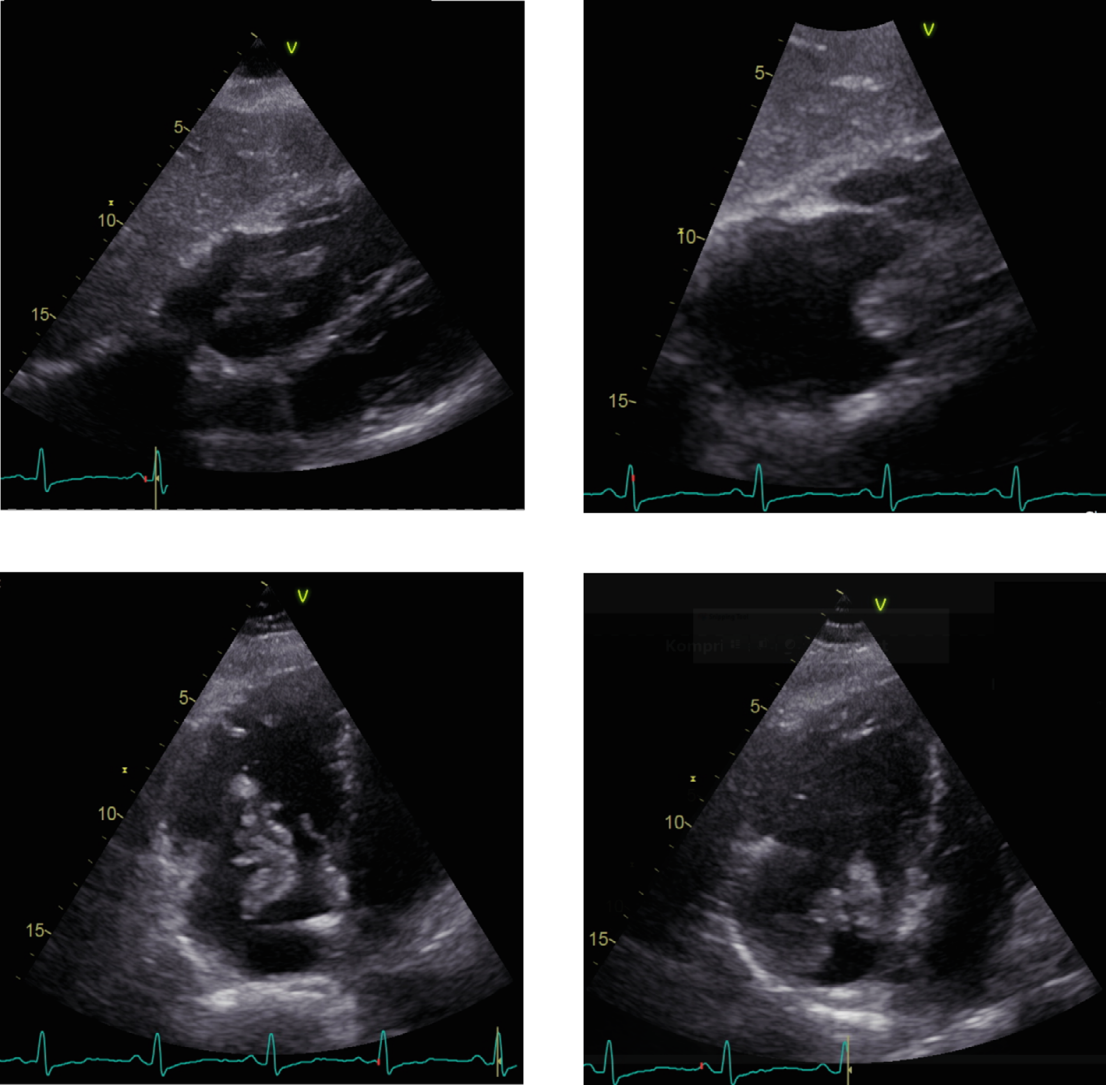
Subcostal and apical four‐chamber view of a mobile thrombus detected in the right heart cavities, originating most likely on the right atrial roof.

Initial investigations included a 12‐lead electrocardiogram (ECG), which showed no new right axis deviation, SIQIII pattern, right bundle branch block, or ST‐segment elevation. Continuous cardiac monitoring revealed no episodes of bradyarrhythmia or tachyarrhythmia. Bedside point‐of‐care transthoracic echocardiography at admission demonstrated a large, highly mobile, filiform thrombus within the right atrium measuring approximately 5 cm in length and 3 cm in width. The thrombus originated from the roof of the right atrium near the cavoatrial junction. With each cardiac cycle, it prolapsed partially into the inferior vena cava and through the tricuspid valve, with about 40% of its length extending into the right ventricle (see Figure 1).

The right ventricle appeared dilated, with a characteristic D‐sign on the parasternal short‐axis view and corresponding interventricular septal flattening. The tricuspid annular plane systolic excursion (TAPSE) measured 18 mm, and the RV S ^′^ velocity was 0.10 m/s, accompanied by a prolonged isovolumic relaxation time (IVRT). Pulmonary artery acceleration time was calculated at 51 ms, showing a slightly notched right ventricular outflow tract (RVOT) curve. The tricuspid valve exhibited moderate regurgitation, and the estimated systolic pulmonary artery pressure (sPAP) was 55 mmHg plus central venous pressure.

The left ventricle appeared small but maintained a preserved systolic function. The left ventricular outflow tract (LVOT) stroke volume was 69 mL, yielding a cardiac output of 6.04 L/min (based on an LVOT diameter of 2.0 cm). The aortic and mitral valves showed no significant abnormalities.

**Figure 2:**
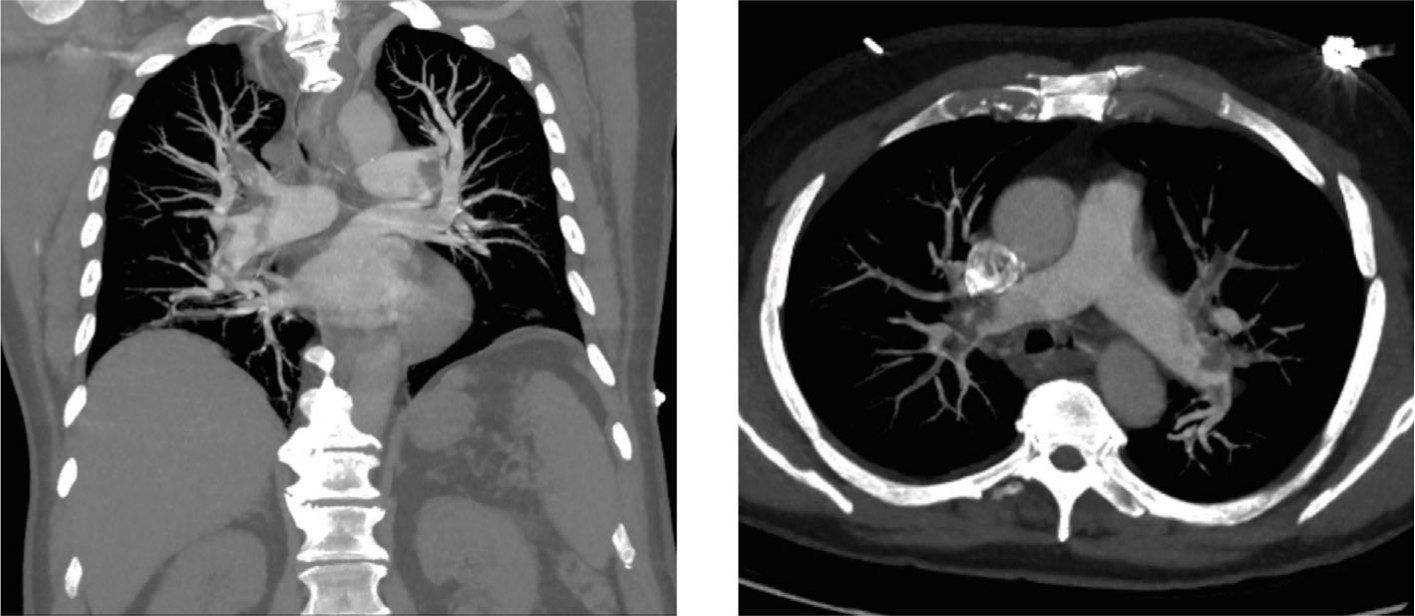
Frontal and sagittal view of bilateral central pulmonary embolism.

Despite the presence of a large thrombus, the patient remained free of resting dyspnea and did not require supplemental oxygen. On admission, troponin levels were elevated to approximately three times the upper reference limit, with only mild to moderate dynamic changes at a 3‐ and 9‐h follow‐up. N‐terminal pro‐B‐type natriuretic peptide (NT‐proBNP) was markedly elevated to 5461 ng/L, indicating significant cardiac strain. Computed tomography pulmonary angiography (CTPA) revealed bilateral central pulmonary emboli with extension into all lobar arteries (see Figure 2). Duplex sonography excluded deep vein thrombosis.

Given the patient’s intermediate–high‐risk profile, unfractionated heparin therapy was initiated, and he was admitted to the intensive care unit for continuous monitoring.

## 3. Considerations and Ongoing Adjustments in Treatment

**Figure 3:**
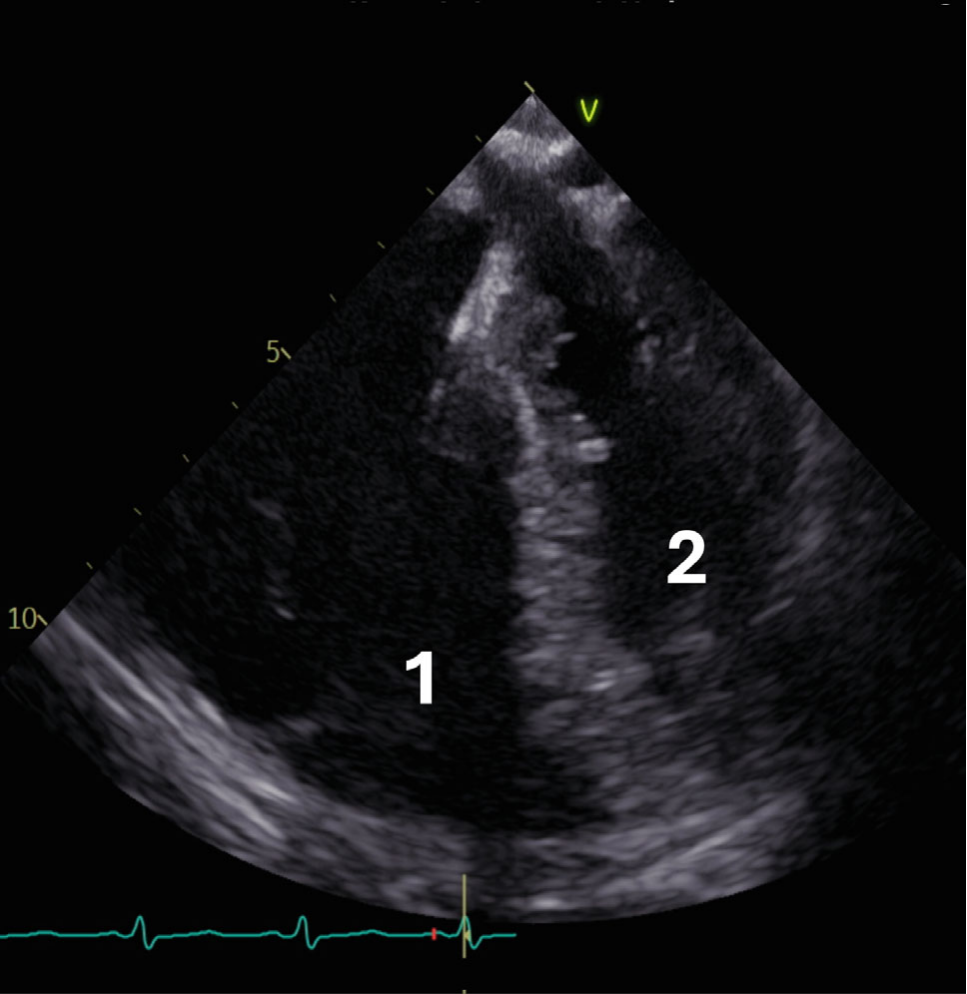
Transesophageal four‐chamber view with no sign of the right atrial or right ventricular (1) thrombus and the left ventricle (2).

As the patient remained haemodynamically stable, management was scheduled by a multidisciplinary team comprising cardiologists, interventional radiologists, and cardiothoracic surgeons. Although the patient fulfilled the criteria for an intermediate‐high–mortality risk according to current guidelines [8], his preserved general condition, moderate bleeding risk, and the substantial hazard posed by a large, potentially major‐vessel–obstructing thrombus rendered a purely conservative strategy inadequate. Given the described high mobility of the thrombus, interventional aspiration was considered unfeasible; therefore, urgent surgical removal under cardiopulmonary bypass was pursued. After obtaining informed consent, preparations for operative intervention commenced. As the patient was admitted over a weekend and resource allocation at our tertiary care center was prioritized according to disease severity, a delay of approximately 24 h occurred between thrombus detection and the scheduled surgical intervention. Bedside echocardiographic assessment showed no change under anticoagulation in the interim. However, upon transfer to the operating theater and induction of anesthesia, routine presurgical transoesophageal echocardiography demonstrated absence of the previously documented right heart thrombus (see Figure 3).

Despite the suspected embolization of the thrombus into the pulmonary arteries, the patient’s hemodynamic status remained stable, leading to the decision to abort the planned surgical intervention. Follow‐up CTPA demonstrated no significant progression of pulmonary artery obstruction. After a comprehensive reassessment of the risk–benefit profile, catheter‐directed therapy was deferred, and systemic thrombolysis was initiated with recombinant tissue‐type plasminogen activator (rt‐PA; alteplase). Therapeutic anticoagulation with unfractionated heparin was maintained thereafter.

The clinical course was complicated by infarct pneumonia, which resolved with targeted antibiotic therapy using ampicillin–sulbactam, combined with respiratory physiotherapy and noninvasive CPAP ventilation via face mask. No further complications occurred. After 10 days, the patient was discharged on a regimen of long‐term anticoagulation.

## 4. Follow‐Up

Subsequent thrombophilia testing, including antiphospholipid antibody screening, was negative except for a heterozygous prothrombin gene mutation. Malignancy was excluded through comprehensive systemic screening. At the 1‐year follow‐up, the patient reported persistent exertional dyspnea, particularly when climbing stairs, requiring two pauses to reach the fourth floor.

Transthoracic echocardiography demonstrated preserved a biventricular systolic function. However, right ventricular morphology remained abnormal, with marked basal dilatation, loss of the characteristic apical configuration, and associated right atrial enlargement. The tricuspid valve was structurally normal and functionally competent.

Cardiopulmonary exercise testing (bicycle ergometry) was terminated at 84 W due to coughing and muscular fatigue. The peak oxygen uptake (VO_2_) was 12.9 mL/kg/min (55% of predicted), consistent with a moderately reduced exercise capacity. The limitation was predominantly circulatory, as indicated by an oxygen pulse plateau, an elevated VE/VCO_2_ (minute ventilation to carbon dioxide output) slope, and a decline in end‐tidal CO_2_ during exertion. NT‐proBNP levels were within normal limits.

Given these findings suggestive of increased pulmonary vascular resistance, chronic thromboembolic pulmonary hypertension (CTEPH) or chronic thromboembolic pulmonary disease (CTEPD; Nice Group 4) was suspected. Further diagnostic evaluation was recommended; however, the patient declined additional testing at that time.

## 5. Discussion

This case illustrates the unpredictable behavior of intracardiac thrombi, particularly those located in the right atrium. The spontaneous disappearance of a right atrial thrombus immediately before surgery is a rare occurrence that can significantly influence the management strategy. Initially, urgent surgical intervention was considered the preferred approach because of the high risk of thromboembolic events, despite the patient’s hemodynamic stability. However, the unexpected resolution of the thrombus permitted a noninvasive therapeutic strategy using systemic thrombolysis, thereby sparing the patient the procedural risks associated with open cardiac surgery.

Given that the clot was free‐floating, the disappearance of the right atrial thrombus can likely be attributed to spontaneous mobilization and embolization. As no thrombolytic therapy had yet been administered, the short interval between the last transthoracic echocardiogram in the intensive care unit and the transesophageal echocardiogram performed in the operating room renders true thrombus dissolution unlikely [9]. However, the minimal hemodynamic impact observed from the suspected embolization remains unusual.

Given the rarity of this occurrence, current clinical guidelines provide no explicit recommendations for patients presenting with concurrent pulmonary embolism and right atrial thrombus. From a clinical standpoint, the Pulmonary Embolism Severity Index (PESI) may underestimate short‐term mortality in this population, as hemodynamic stability can rapidly deteriorate with progressive pulmonary artery obstruction due to recurrent embolization. An individualized, dynamic approach to management is therefore warranted to account for the elevated risk and unpredictable clinical course.

This case underscores the critical importance of multidisciplinary collaboration, prompt decision‐making in life‐threatening scenarios, and continuous re‐evaluation of the patient’s condition with readiness to adapt therapeutic strategies to evolving circumstances.

## 6. Learning Points

- Transthoracic echocardiography is valuable for rapid, noninvasive diagnosis in patients with presyncope symptoms. A three‐window protocol (parasternal short axis, parasternal long axis, and apical four‐chamber) might sufficiently identify high‐risk patients.
- A free‐floating thrombus in the right atrium carries a significant risk of dislodgement, potentially leading to a life‐threatening pulmonary embolism.
- Routine ultrasound evaluation immediately before surgical treatment of highly mobile thrombi is crucial for confirming the necessity and appropriateness of intervention.
- Thrombophilia screening is essential for patients at increased risk of thromboembolic events.

## Consent

The authors affirm that the patient has provided written informed consent for the submission and publication of this case report, including all associated images and text, in compliance with COPE guidelines.

## Disclosure

E.H. received educational and travel support from Bayer, Boston Scientific, Edwards Lifesciences, Medtronic, and Pfizer, unrelated to the submitted work.

## Conflicts of Interest

The authors declare no conflicts of interest.

## Funding

Open Access funding enabled and organized by Projekt DEAL.

## Data Availability

The data underlying this article will be shared on reasonable request to the corresponding author.
